# Complement in malaria: immune evasion strategies and role in protective immunity

**DOI:** 10.1002/1873-3468.13772

**Published:** 2020-04-01

**Authors:** Patience Kerubo Kiyuka, Seppo Meri, Ayman Khattab

**Affiliations:** ^1^ Department of Bacteriology and Immunology Translational Immunology Research Program Haartman Institute University of Helsinki Finland; ^2^ KEMRI‐Wellcome Trust Research Programme Centre for Geographic Medicine Research‐Coast Kilifi Kenya; ^3^ Helsinki University Central Hospital Finland; ^4^ Department of Biomedical Sciences Humanitas University Milan Italy; ^5^ Department of Nucleic Acid Research Genetic Engineering and Biotechnology Research Institute City of Scientific Research and Technological Applications Alexandria Egypt

**Keywords:** complement, immunity, malaria, *Plasmodium*

## Abstract

The malaria parasite has for long been thought to escape host complement attack as a survival strategy. However, it was only recently that complement evasion mechanisms of the parasite were described. Simultaneously, the role of complement in antibody‐mediated naturally acquired and vaccine‐induced protection against malaria has also been reported. Such findings should be considered in future vaccine design, given the current need to develop more efficacious vaccines against malaria. Parasite antigens derived from molecules mediating functions crucial for parasite survival, such as complement evasion, or parasite antigens against which antibody responses lead to an efficient complement attack could present new candidates for vaccines. In this review, we discuss recent findings on complement evasion by the malaria parasites and the emerging role of complement in antibody‐mediated protection against malaria. We emphasize that immune responses to vaccines based on complement inhibitors should not only induce complement‐activating antibodies but also neutralize the escape mechanisms of the parasite.

## Graphical abstract

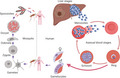

## Abbreviations

**C1‐INH**, C1‐inhibitor

**C3bBb**, C3 convertase

**C4BP**, C4b‐binding protein

**CCP**, complement control protein

**CR1**, complement receptor 1

**CRIg**, complement receptor of immunoglobulin

**CSP**, circumsporozoite protein

**DAF**, decay‐accelerating factor

**FH**, factor H

**FHbp**, factor H‐binding protein

**MAC**, membrane attack complex

**MASP2**, mannan‐binding lectin‐associated serine protease 2

**MBL**, mannose‐binding lectin

**MCP**, membrane cofactor protein

**NAI**, naturally acquired immunity

**PfEMP1**, *P. falciparum* erythrocyte membrane protein 1

**PfGPI**, *P. falciparum* glycosylphosphatidylinositol

**VSA**, variant surface antigen

**Fig. 1:**
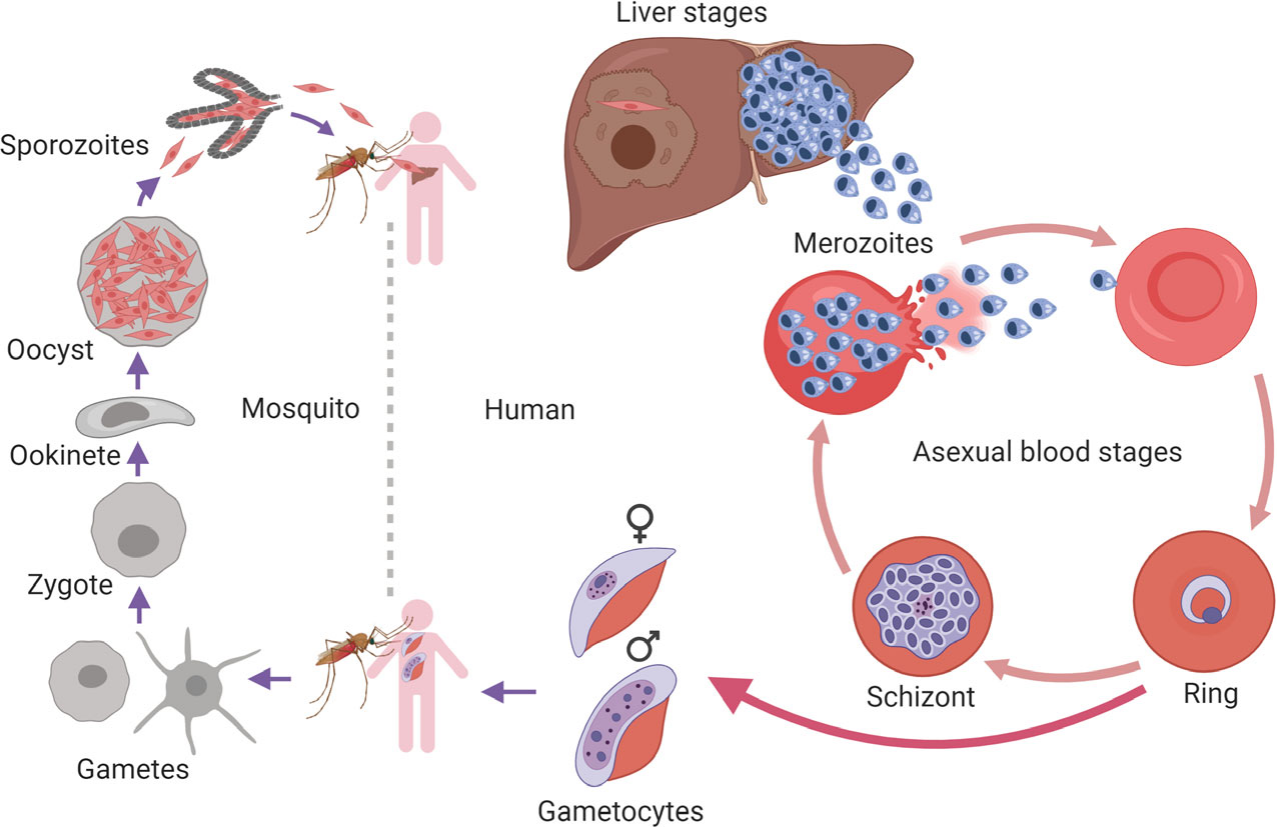
Life cycle of the malaria parasite *P. falciparum*.

The global burden of malaria is massive. In 2018 alone, an estimated 228 million cases occurred globally. There were 405 000 deaths reported; 67% occurred in children under the age of 5 years. Of all malaria deaths, 93% occurred in sub‐Saharan Africa [1]. Malaria in humans is caused by five species of *Plasmodium*: *P. falciparum*, *P. vivax*, *P. malariae, P. ovale*, and *P. knowlesi*. *P. falciparum* causes the most severe forms of malaria. The life cycle of *Plasmodium* parasites is complex (Fig. 1) [2]. They require an anopheline mosquito vector and a vertebrate host [3]. In the human host, infection begins with the bite of an infected *Anopheles* mosquito that inoculates the person with about 10–100 sporozoites [4]. The sporozoites migrate to the capillaries and then travel *via* the bloodstream into the liver, where they invade hepatocytes [5] and initiate the pre‐erythrocytic cycle [6]. Upon invasion of hepatocytes, the parasites undergo replication, also called schizogony, at the end of which thousands of daughter cells are formed. These cells differentiate into invasive blood‐stage parasites called merozoites that are released in ‘batches’ into the bloodstream [7]. In the bloodstream, the merozoites quickly invade erythrocytes to initiate the pathogenic erythrocytic stage of the parasite life cycle, also called the asexual cycle. Within the erythrocytes, the merozoites mature to form rings, then trophozoites, and eventually schizonts with multiple parasites that burst to release a new generation of merozoites, thereby starting the process of invading new erythrocytes [8]. The asexual life cycle is accompanied by commitment to gametocytogenesis [9, 10]. Thereby, a fraction of merozoites differentiate inside erythrocytes into male and female gametocytes [11] that are taken up during a mosquito’s blood meal. In the mosquito midgut, male and female gametes fuse to form ookinetes that cross the midgut epithelium, where they develop into oocysts. Parasites inside oocysts differentiate ultimately into sporozoites that invade the mosquito’s salivary glands [12].

The complex life cycle of malaria offers multiple potential points of intervention. Malaria vaccine development has primarily focused on pre‐erythrocytic, blood‐stage, or transmission‐blocking vaccine targets [13]. However, after more than 50 years of intensive research and development, only one malaria vaccine candidate, RTS,S (Mosquirix®), based on the central repeat and C‐terminal epitopes of the major sporozoite surface antigen, circumsporozoite protein (CSP), have completed phase‐3 trials. Results of these trials indicated that this vaccine reduced episodes of clinical malaria in children 5–17 months and infants 6–12 weeks of age by 50.4% and 30.1%, respectively, over a 1‐year follow‐up [14]. Despite this positive outlook, it was apparent that the vaccine was only partially protective, and the obtained immunity waned over time, as the RTS,S vaccine efficacy was only 28% in children 5–17 months of age and 18% in infants 6–12 weeks of age when measured 2 years after vaccination [15, 16]. Therefore, innovative approaches are urgently needed to design vaccines with improved efficacies [17].

The complement system represents a major part of the innate immunity and is considered the first line of defense against pathogens. It is a cascade of soluble plasma proteins and membrane‐expressed receptors and regulators, which operate in plasma and other body fluids, in tissues and on cell surfaces. Recent studies demonstrated that *P. falciparum* parasite has surface molecules that can capture host soluble complement regulators to inhibit complement activities and avoid cell damage [18, 19, 20]. Other studies described a role for complement in antibody‐mediated protection against malaria [21, 22, 23, 24, 25, 26, 27]. These findings could be exploited to design a malaria vaccine that can induce antibodies with an ability to neutralize complement evasion mechanisms and activate the complement, thus resulting in enhanced complement‐mediated protection.

In the current review, we will discuss recent findings on complement evasion by the *P. falciparum* and the emerging role of the host complement system in antibody‐mediated protection against malaria. We will also emphasize that immune responses to vaccines based on complement inhibitors should not only induce antibodies that activate the complement (complement‐fixing antibodies) but also neutralize the escape mechanisms of the parasite.

## The complement system activation pathways and regulation

**Fig. 2:**
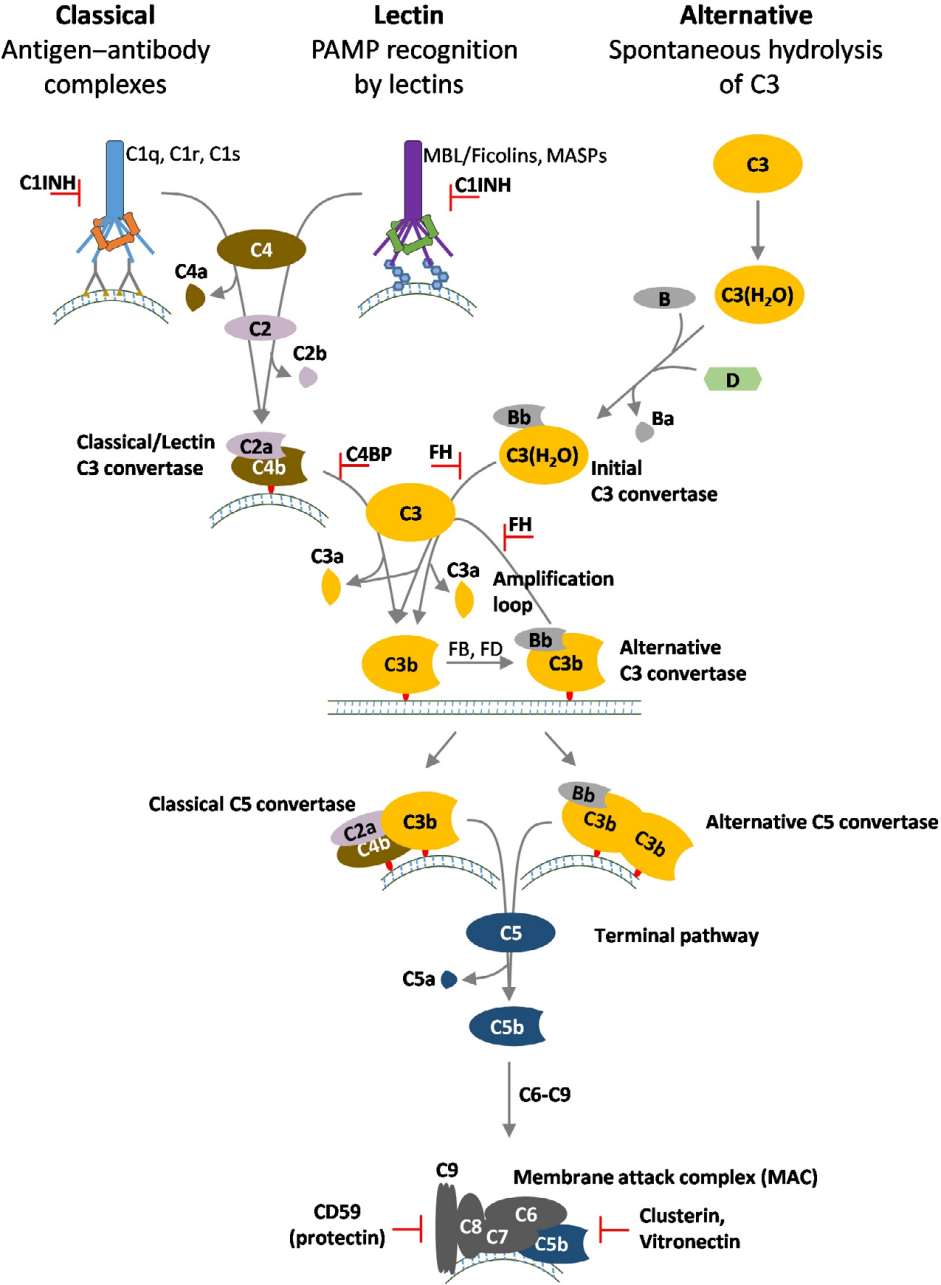
Pathways of complement activation. The complement system forms the first line of defense against invading pathogens. It can be activated through three major pathways: the classical pathway, the lectin pathway, and the alternative pathway. Antigen–antibody complexes are recognized by C1q of the classical pathway, MBL and ficolins binding to foreign surfaces activate the lectin pathway, while spontaneous hydrolysis of native C3 will initiate the alternative pathway. Upon activation, there is initial deposition of C3b on the foreign surface, which will generate the actual alternative pathway C3bBb that boosts a feedback amplification loop. Through the formation of C3bBb, all pathways culminate in the formation of C3b and the anaphylatoxin C3a. Subsequent C5 convertase formation leads to C5b and anaphylatoxin C5a generation, with C5b initiating the formation of the MAC, which becomes inserted into target cell membranes. Host tissues and cells are protected from complement deposition by fluid‐phase and cell‐bound regulators. C1‐INH inhibits the functions of C1r, C1s, and MASP2. C3b (and C4b) is inactivated by complement factor I and one of several cofactor proteins (surface‐bound CD46 and complement receptor type 1 (CR1) or fluid‐phase factor H or C4BP). Convertases are regulated through disassembly by regulators that have decay‐accelerating activity (CD55, CR1, factor H, and C4BP). The formation of the MAC is controlled by the activities of CD59, clusterin, and vitronectin.

The complement system forms part of the innate immune response and serves as an effector arm of adaptive humoral immunity. Its key functions include C1q‐, C3b‐, and iC3b‐mediated cell opsonization for phagocytosis, immune cell recruitment, and induction of inflammation mediated by release of C3a and C5a and formation of the membrane attack complex (MAC) for targeted microbial lysis [28]. There are three main complement pathways: the classical pathway, the lectin pathway, and the alternative pathway (Fig. 2). The classical pathway is triggered by the recognition of antibody–antigen complexes on foreign cell surfaces by complement component C1q. Structurally similar pattern‐recognition receptors, mannose‐binding lectin (MBL) and ficolins, bind to carbohydrate ligands on microbial intruders and initiate the lectin pathway. On the contrary, the alternative pathway is initiated by the spontaneous hydrolysis of native C3 and the absence of self‐surface structures. Activation of each of these pathways results in the assembly of critical enzymes of the cascade called C3 convertases (C3bBb). Once activated, all the three pathways converge at the C3 step, leading to covalent deposition of the opsonins C3b and iC3b, generation of inflammatory anaphylatoxins C3a and C5a, and ultimately the formation of MAC [29].

**Fig. 3:**
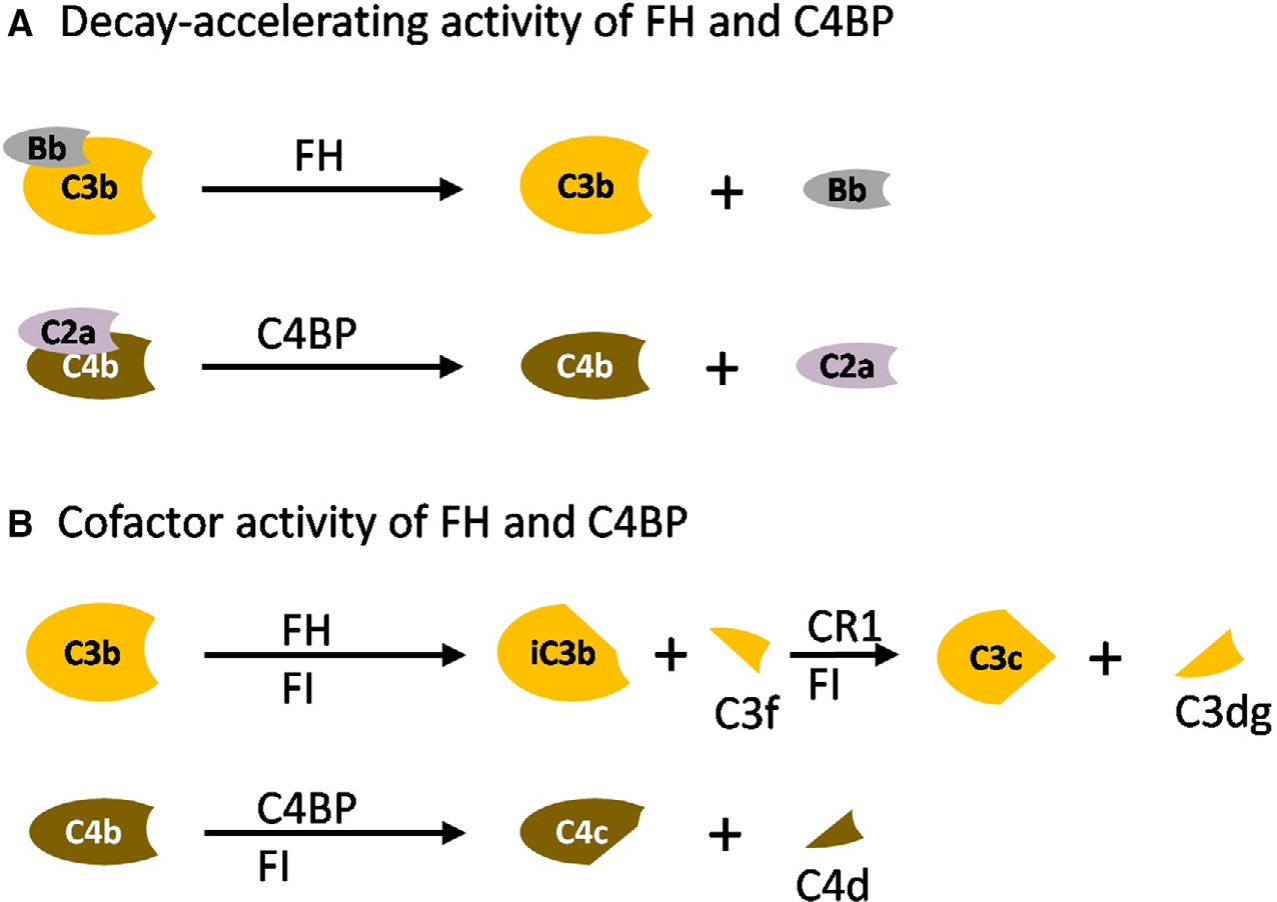
Regulation of complement activity by the soluble regulators FH and C4BP. (A) FH and C4BP have decay‐accelerating activities for the alternative and classical C3bBb, respectively. (B) FH and C4BP act as cofactors promoting FI‐mediated cleavage of C3b and C4b.

The complement system is tightly controlled by fluid‐phase regulators [e.g., factor H (FH), factor H‐like protein 1 (FHL‐1), C4b‐binding protein (C4BP), and C1‐inhibitor (C1‐INH)] and by cell membrane regulators [e.g., complement receptor 1 (CR1)/CD35, MCP/CD46, decay‐accelerating factor (DAF)/CD55, complement receptor of immunoglobulin (CRIg), and protectin/CD59] to prevent inappropriate complement activation and host cell destruction [30]**.** Complement receptor type 1 (CR1/CD35), membrane cofactor protein (MCP/CD46), DAF/CD55, and CRIg inhibit complement activation at the C3bBb level. Protectin/CD59 prevents complement activation by binding to C5b‐8 and C5b‐9 complexes, thereby preventing C9 from polymerizing and forming the MAC [31]. C1‐INH and C4BP inhibit the initial steps of the classical pathway. Specifically, C1‐INH inhibits the functions of C1r, C1s, and mannan‐binding lectin‐associated serine protease 2 (MASP2). Clusterin and vitronectin inhibit the terminal pathway. FH and FHL‐1, an alternatively spliced product of the factor H gene, regulate the amplification loop of the alternative pathway [32]. The functions of FH include dissociation of the C3bBb, also called decay‐accelerating activity, and cofactor activity for factor I‐mediated cleavage and inactivation of C3b (Fig. 3).

## Complement inhibition by microbes

**Fig. 4:**
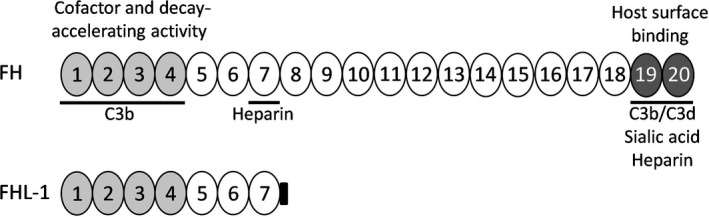
Schematic representation of the CCP domains of FH (1–20) and FHL‐1 (1–7). Binding sites for C3b, C3d, sialic acid, and heparin are shown by horizontal lines. FHL‐1 is a splice variant of FH.

Many immune evasion strategies developed by pathogens against innate immunity are focused on the complement system. Pathogens utilize multiple strategies to interfere with complement immune recognition and effector functions in order to survive in the human host [33, 34, 35]. They express diverse and multiple surface proteins and secrete additional molecules to avoid complement attack. Commonly shared strategies include interference at the activation or at the C3 and C5 convertase level and inhibition of MAC formation. Perhaps the most commonly studied complement‐inhibiting mechanism of pathogens is the recruitment of complement regulatory protein FH [36]. Pathogens commonly recruit FH to their surfaces by binding to complement control protein (CCP) domains 6–7 or CCP 19–20 leaving the critical regulatory region of FH CCP1‐4 functional. Bacterial proteins that bind FH *via* CCP 6–7 can also recruit FHL‐1 due to the shared conserved CCP domains between FH and FHL‐1 (Fig. 4) [37]. Examples include outer surface protein E for *Borrelia burgdorferi* [38], staphylococcal binder of immunoglobulin for *Staphylococcus aureus*, pneumococcal surface protein C for *Streptococcus pneumoniae* [39], and factor H‐binding protein (FHbp) for *Neisseria meningitidis* [40], all of which recruit factor H [41].

Interestingly, FHbp proteins are now components of Bexsero® (Novartis, Basel, Switzerland, one FHbp protein) and Trumenba® (Pfizer, Newyork, NY, USA, two FHbp proteins), two recently approved vaccines against group B meningococcus [42]. In addition to activating complement, FHbp‐specific antibodies block binding of FH and hence increase bacterial susceptibility to killing *via* the alternative pathway. Although complement evasion mechanisms and molecules have been identified for several pathogens during the past decades, it was only recently that evasion mechanisms and molecules have been identified for the *P. falciparum* parasite [19, 20, 43, 44].

## Complement immune evasion at the different stages of the *Plasmodium* parasite

### Sporozoites

Exposure of the developmental stages of the malaria parasite to human complement within the complex life cycle of the parasite is clearly evident. The invasive extracellular sporozoites that are inoculated *via* a mosquito bite into human skin provide one example. Sporozoites take about 20 min from the inoculation site to reach the liver. They are exposed to complement yet succeed in infecting hepatocytes. This suggests that sporozoites can escape complement effector functions. For *P. falciparum,* this is probably also host species‐specific, because it does not infect other vertebrates in nature.

Indeed, there are reports on the resistance of malaria sporozoites to complement. Data from *Plasmodium berghei* show that in the presence of nonhost species human serum, salivary gland sporozoites activate complement causing deposition of C3b. Furthermore, the C3b deposition led to a reduction in sporozoite infectivity to HepG2 cells. Conversely, loss of infectivity to HepG2 cells did not occur when the sporozoites were treated with serum from the susceptible host (mouse). Additionally, after shaving off their surface with trypsin, *P. berghei* sporozoites became the target for complement in the serum of susceptible hosts and deposited C3b on their surfaces [45]. Moreover, Touray *et al*. [46] reported differences in the susceptibility to complement lysis between two developmentally distinct sporozoite stages that were isolated during the sporogenic development of *P. gallinaceum* in the mosquito vector. It was found that the *P. gallinaceum* noninfectious oocyst sporozoites were susceptible to host (chicken) complement lysis, whereas the highly infectious salivary gland sporozoites were resistant to complement lysis. Because oocyst sporozoite lysis by serum was heat‐sensitive (+56 °C) and Mg^2+^‐dependent, but Ca^2+^‐independent, the authors concluded that lysis was mediated by the alternative pathway of complement [46]. Data on the resistance of sporozoites to complement lysis also suggest that they express proteins on their surfaces to interfere with complement activity [45]. Thus far, no evasion mechanisms have yet been discovered for *P. falciparum* sporozoites, which represents a major knowledge gap in the field.

### Merozoites

**Fig. 5:**
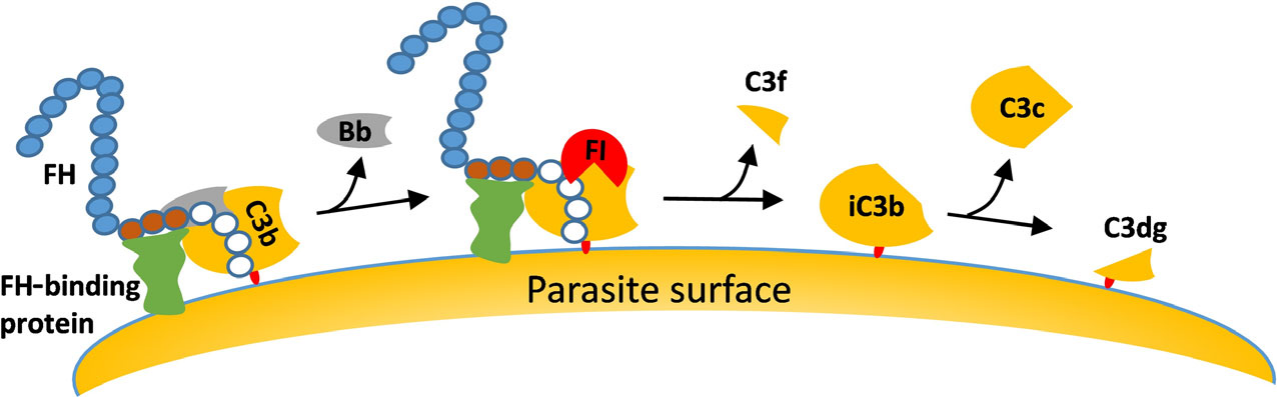
Model of complement evasion on parasite surface through acquisition of the soluble host complement regulator FH.

The invasive extracellular merozoites that are released from the erythrocytic‐stage schizont of the malaria parasite into the bloodstream to invade new erythrocytes are another clear example of exposure to the complement system. Within the human host, there is evidence that intraerythrocytic schizonts and free merozoites bind FH and FHL‐1 to their surfaces. This binding inactivates C3b (depicted in Fig. 5) to ensure survival during the erythrocytic replication phase [44]. Indeed, of all the parasite asexual stages, free merozoites and intracellular mature schizonts have been shown to significantly bind more FH molecules than trophozoites and rings [43]. So far, only merozoite protein Pf92 has been characterized as the protein responsible for interaction with FH [19]. Pf92 was also shown to bind to the CCP domains 5–6 of FH and FHL‐1. The deletion of the Pf92 gene led to somewhat increased complement‐mediated destruction of merozoites [19]

It has been demonstrated that merozoite proteins actively recruit C1‐INH to their surfaces, allowing the parasite to limit the classical pathway proteases and hence avoid downstream complement activation [20]. PfMSP3.1 was identified as the C1‐INH interacting partner. Interestingly, although PfMSP3.1 parasite knock‐outs showed reduced C1‐INH recruitment and increased C3b deposition, which would imply that they were more susceptible to complement‐mediated killing, the knock‐out parasite paradoxically had enhanced erythrocyte invasion in the presence of active complement [20].

Merozoites have also been reported to bind C1‐INH to their surface through an interaction with glycan moieties within the *P. falciparum* glycosylphosphatidylinositol (*Pf*GPI) molecule [47]. Merozoite binding of increasing concentrations, up to fivefold higher than serum normal level, of C1‐INH inhibited invasion of erythrocytes in the absence of active complement. Thus, it was postulated that binding of C1‐INH to GPIs at the merozoite surface may have prevented the molecular processes associated with erythrocyte invasion that involve recognition of erythrocytes by parasite ligands, including GPI‐anchored parasite proteins. Together, these results demonstrate that binding of C1‐INH to the merozoite surface could be either beneficial or detrimental to the parasite. Therefore, the role of C1‐INH binding as a parasite complement evasion mechanism still needs further investigation.

Potentially, CR1 on erythrocytes could also contribute to complement escape by malaria parasites, provided that it becomes released and binds to merozoites.

### Asexual erythrocytic stages

As a result of invasion of erythrocytes, *P. falciparum* parasites start immediately to remodel and modify the host cells, leading to dramatic structural and morphological changes on their surfaces [48]. Among these changes is the export of parasite proteins to the surface of erythrocytes [49] to mediate cytoadhesion and sequestration of the infected erythrocytes (IE) in the microvasculature to avoid clearance by the spleen [50]. If the adhesion proteins are conserved, the immune system may react toward them and recognize them in subsequent infections and destroy the parasite. This is obviously not the case; the parasite has developed a means to evade the immune recognition by varying the parasite’s exposed surface proteins [50]. It is now well established that the *P. falciparum* erythrocyte membrane protein 1 (PfEMP1), encoded by ~ 60 copies of the highly polymorphic *var* genes, undergoes antigenic variation and is responsible for adhesion to various host cell receptors [51, 52]. Adults in areas where malaria is endemic are normally clinically immune to the disease. This is a result of repeated infections with *P. falciparum* [53, 54] and the concurrent exposure to variant surface antigen (VSA), of which PfEMP1 is the most prominent and best studied. Variant‐specific immunity is also acquired in an age‐dependent manner [55, 56, 57] and associated with protection from clinical disease [55, 58]. Since activation of the classical pathway of complement relies on binding of C1q to antibody–antigen complexes, antigenic variation on the *P. falciparum‐* IE surface would limit complement fixation and cell lysis. Thus, it could be considered as a complement evasion mechanism, at least in individuals with limited exposure to malaria, that is, with a limited repertoire of anti‐VSA antibodies. However, early work showed that immune serum (with a large anti‐VSA antibody repertoire) from malaria‐exposed donors fixed complement on *P. falciparum* IE surface, but no cell lysis [59] nor other adverse effects on parasite development [60] were observed. This indicated that mechanisms other than antigenic variation have contributed to *P. falciparum* IE resistance to complement‐mediated cell lysis. Wiesner *et al*. [60] have demonstrated that the absence of functional endogenous CD59, responsible for preventing the formation of MAC, on the erythrocyte surface rendered *P. falciparum* IE susceptible to complement lysis. Hence, one obvious mechanism for the observed complement resistance of IE is mediated by the host cell‐intrinsic complement regulator CD59.

On the other hand, a recent study by Larsen *et al*. [61] demonstrated that polyclonal and a monoclonal human IgG targeting a specific variant of PfEMP1 were not able to activate complement on *P. falciparum* IE that expressed this specific variant even though these antibodies fixed complement in an ELISA format assay when bound to the recombinant PfEMP1. The study authors concluded that the knob‐restricted display (clustering in knob‐like protrusions on the erythrocyte membrane) of PfEMP1 interfered with the on‐target hexamerization of IgG and prevented complement fixation [61]. For IgG to efficiently activate complement and lead to target cell lysis, it has to deposit densely enough on the target cell to accommodate C1q binding. IgG also has to bind close to the target cell membrane, so that C3bBbs and MAC can be targeted to the membrane. Molecules that activate complement at distance like IgG binding to the tips of surface molecules will in fact divert complement attack away from the target. Finally, it is also important that the antibodies block complement inhibitors on the target. Otherwise, a clearly larger number of IgG molecules need to be deposited to overcome the inhibitory effect of these molecules. The net result will thus depend on the balance of complement‐activating and complement‐inhibiting potential on the target.

Additionally, another variant of PfEMP1 was also found to bind human nonimmune IgM [62] and occupy C1q‐binding sites on IgM and hence prevent complement fixation [63]. Thus, both the knob‐restricted display of PfEMP1 and blocking C1q‐binding site on IgM could be considered as parasite’s strategies to evade acquired protective immunity and complement activation. However, data discrepancy on complement fixation on the surface of *P. falciparum* IE justifies the need for further investigations to better understand complement evasion by blood‐stage *P. falciparum* parasites.

### Gametes

When a mosquito feeds on an infective host, malaria parasite sexual stages (female and male gametes) enter the mosquito midgut where gametogenesis is induced and gametes emerge from the erythrocytes. Once they emerge, they are exposed to the active immune components of the host blood [64]. In fact, complement proteins factor B, factor D, and C3, components of the alternative pathway of complement activation, remain active for several hours in the mosquito midgut [65, 66]. It is during this mosquito stage of development that *P. falciparum* gametes recruit FH and FHL‐1 (from the human blood ingested during a blood meal) to their surfaces to evade human complement attack within the mosquito midgut [18]. The gamete surface protein GAP50 binds FH and uses it to inactivate the complement protein C3b. The binding site of GAP50 was mapped to CCP modules 5–7 of FH and FHL‐1 [18]. Furthermore, polyclonal antibodies directed against GAP50, when taken with the infected blood meal, only partially blocked oocyst formation, which could indicate that GAP50 was not the sole FH parasite binding partner [18]. Moreover, the *Anopheles* mosquito midgut cells have been shown to protect themselves from human complement lysis by also capturing FH [66]. Khattab *et al*. [66] also demonstrated that the captured FH promoted inactivation of formed C3b molecules on the midgut epithelium and limited formation of the C3bBb enzymes. A hitherto putative FH receptor protein on the midgut cells was identified, and further work is required to characterize it [66]. On the other hand, the chicken malaria parasite *P. gallinaceum* zygotes have been shown to be susceptible to the alternative pathway destruction in the presence of nonhost (human, sheep, and guinea pig) serum *in* *vitro*. In the natural chicken host, however, trypsin‐sensitive surface proteins conferred protection against the alternative complement pathway [67]. Furthermore, although *P. berghei* macrogametes’ and zygotes’ resistance to complement has been investigated [65] neither the complement regulatory protein nor its receptor responsible has been conclusively identified.

**Fig. 6:**
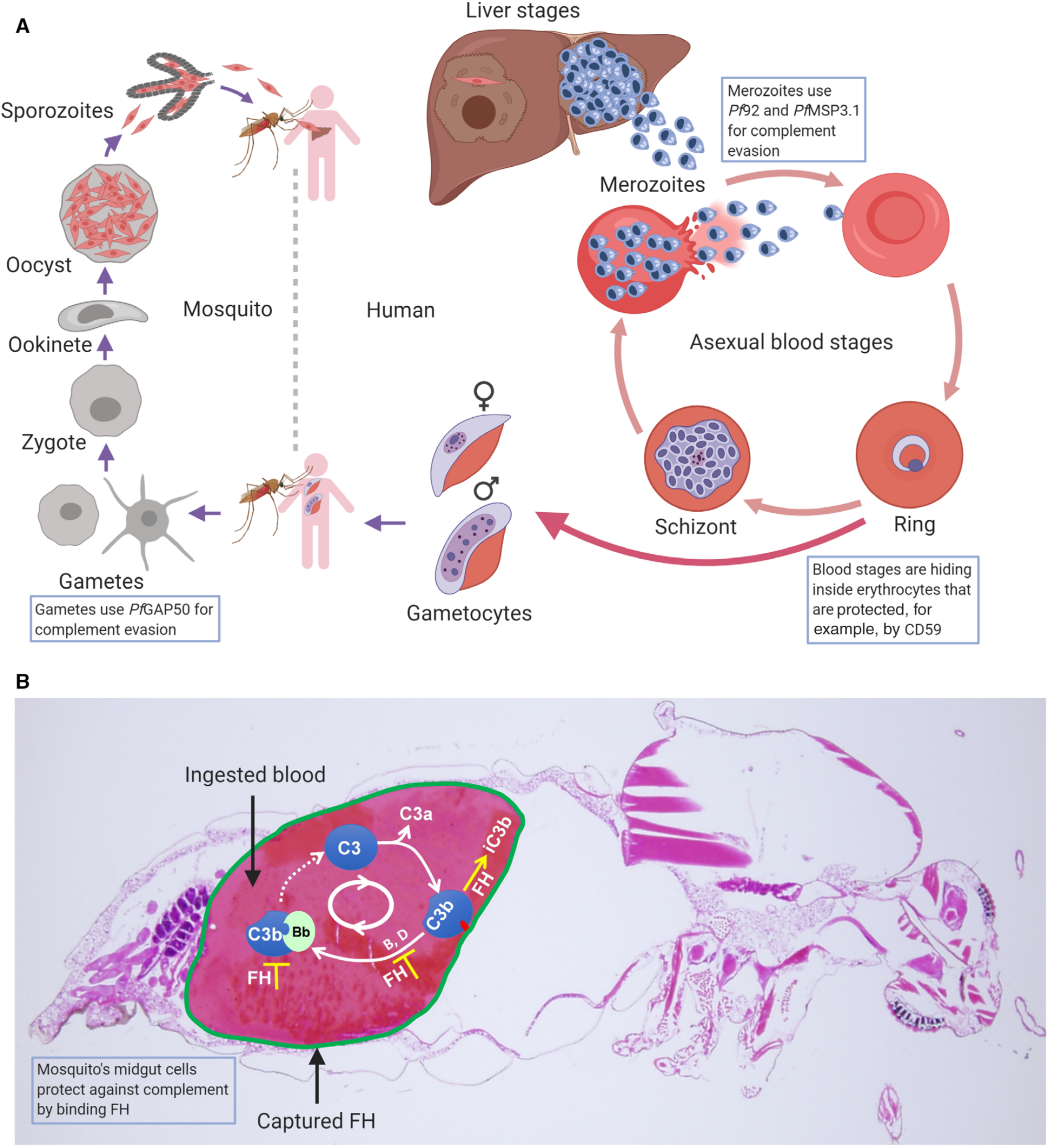
Involvement of complement in the *P. falciparum* life cycle. (A) The life cycle of the *P. falciparum* parasite in mosquito and human (created with BioRender.com). Boxes highlight the developmental stages at which complement is inhibited by parasite molecules or provides protection to the parasite, when it is residing inside erythrocytes. (B) Sagittal section of a blood‐engorged Anopheles mosquito stained with hematoxylin and eosin (created in the senior author’s laboratory). Spontaneous hydrolysis of native C3 activates the alternative pathway. Upon activation, there is initial deposition of C3b on the midgut epithelium, which can also initiate a feedback amplification loop. Captured FH on the midgut epithelium acts as an accelerator of the decay of C3bBb and as a cofactor for factor I in the proteolytic inactivation of C3b to iC3b.

In summary, complement evasion mechanisms have been described in the *P. falciparum* life cycle for the merozoite and gamete stages, as well as for the mosquito midgut cells. These evasion mechanisms are highlighted on the parasite life cycle in Fig. 6. Other findings suggest the resistance of the sporozoite and the erythrocytic stages of the parasite to complement attack, but no precise evasion mechanisms have yet been described.

## Role of complement in naturally acquired protection against malaria

More than 110 years ago, Robert Koch demonstrated that individuals living in malaria‐endemic areas develop naturally acquired immunity (NAI) to malaria. Koch based his observations on microscopically detectable parasitemia in children, adults, and transmigrants in highly endemic areas of Papua New Guinea and Indonesia, where people were exposed to many infectious bites each year. By observing higher rates of adults with asymptomatic infections and higher rates of children with symptomatic infections, he concluded that immunity develops slowly after many years of exposure, but that complete immunity was never really achieved [68]. Years later, the essential features of Naturally acquired immunity have been described. It is now generally accepted that natural immunity to malaria was (a) effective in adults after uninterrupted lifelong continuous exposure, (b) lost upon cessation of exposure, (c) parasite species‐specific, (d) somewhat stage‐specific, and (e) acquired at a rate dependent upon the degree of exposure [69]. Antibodies are known to be important components of NAI to malaria. The strongest evidence came from studies of Cohen *et al*. [70], in which passive transfer of purified IgG from immune African adults to children admitted with clinical malaria was shown to significantly reduce parasitemia and lead to the resolution of fever [70]. Serum antibodies, which are made by plasma cells, mediate protection by acting predominantly against parasites of the asexual blood stages that cause the clinical symptoms of malaria [71]. However, a knowledge gap remains on which specific antigens induce protective immunity, and which antibody effector functions are the most important ones in protection from disease.

Thus, efforts to develop a highly efficacious malaria vaccine have been hampered by (a) the limited understanding of functional mechanisms that contribute to protection against malaria and by (b) the insufficient knowledge of correlates of protection. Nevertheless, significant progress has been made in defining mechanisms of antibody‐mediated immunity [72, 73]. Previously, it was known that antibodies could function in conjunction with phagocytic cells (opsonophagocytosis) [74], stimulate monocytes and macrophages to release molecules that kill parasites (antibody‐dependent cellular cytotoxicity) [75], or directly inhibit parasite invasion, also called growth inhibition [76]. There is now a significant body of literature suggesting that complement is important in antibody‐mediated immunity. It has been shown that complement is involved in the pre‐erythrocytic‐, blood‐, and sexual‐stage immunity against malaria parasites.

## Complement in immunity to pre‐erythrocytic stages

Immuno‐epidemiological studies from malaria‐endemic areas have shown that individuals continuously exposed to malaria can develop antisporozoite antibodies (including those targeting CSP) [77, 78, 79]. However, there has been no significant association between the acquisition of antibodies against CSP and protection from clinical malaria [80, 81]. Nonetheless, a recent study by Kurtovic *et al*. [21] has shown that anti‐CSP antibodies from malaria‐exposed populations that were predominately IgG1, IgG3, and IgM can fix complement and hence activate the classical pathway. These antibodies also inhibited sporozoite traversal of hepatocytes and caused sporozoite death *in* *vitro*. Additionally, high levels of the complement‐fixing antibodies were significantly associated with protection against clinical malaria in children. However, it was observed that complement‐fixing antibodies were acquired more slowly to sporozoite antigens than to merozoite antigens [21]. This finding could be simply attributed to the fewer number of sporozoites that the immune system encounters in an infection.

## Complement in immunity to merozoites

It is well established that antibodies play an important role in blood‐stage immunity [70]. Acquired and vaccine‐induced human antibodies recruit complement and interfere with erythrocyte invasion by the malaria parasite [82]. Boyle *et al.* [82] have described an antibody effector function known as antibody‐mediated complement‐dependent (Ab‐C’) inhibition, in which antibodies use complement to inhibit merozoite invasion. In some individuals, the antibodies were shown to be noninhibitory in the absence of complement. Antibodies that fix C1q were shown to be associated with protection from clinical episodes of malaria [82]. The importance of complement is further supported by the association of protection from malaria with levels of cytophilic IgG1 and IgG3 that activate complement *via* C1q [83, 84, 85]. Similarly, merozoite‐specific IgM antibodies were identified in an experimental exposure and in naturally acquired malaria cohorts and shown to inhibit merozoite invasion of erythrocytes in a complement‐dependent manner [24]. Moreover, the merozoite‐specific IgM antibody response was long‐lived and associated with protection in a longitudinal cohort of naturally exposed children [24]. These data suggest that IgM responses, alongside with IgG and in a complement‐dependent manner, are an important contributor to NAI against malaria [24]

Perhaps a significant step forward in our understanding of the antibody–complement effector function is that the specific targets for complement‐fixing antibodies are now known [25]. The recent work of Reiling *et al.* [25] has shown that EBA140 RIII‐V, RASPI, GAMA, PfRH2, MSP‐DBL1, PfRH5, EBA 175‐RIII‐V, and MSP2‐3D7 merozoite proteins are targets for complement‐fixing antibodies. Importantly, antigen‐specific complement‐fixing antibodies were strongly associated with protection from malaria in a longitudinal study of children. Also, using statistical modeling, they observed that combining complement‐fixing antibody responses to three antigen targets could increase the potential protective effect to over 95%. These findings support antibody–complement interactions against merozoite antigens as important antimalarial immune mechanisms [25]. In contrast, Biryukov *et al.* [86] have shown that antimerozoite antibodies and complement activation can paradoxically aid the malaria parasite to invade erythrocytes. Using a monoclonal antibody directed against the merozoites and human polyclonal IgG from merozoite vaccine recipients in a standard invasion inhibition assay, they showed that in the presence of complement and antibodies, there was enhanced invasion [86]. However, unlike the previous studies, this study used naturally egressed merozoites rather than filter‐purified merozoites [82]. Moreover, Biryukov *et al*. [86] showed that filter‐purified merozoites appeared less infective and more sensitive to complement activation than the naturally egressed merozoites. The advantage of using filter‐purified merozoites would be (a) to have significantly less parasite debris in the assay and (b) to avoid the time window needed for the release of merozoites from mature schizonts (in the naturally egressed merozoite assays). Both of these mechanisms could otherwise activate complement in the liquid phase and lead to undesired consumption of complement. On the other hand, naturally egressed merozoites would be healthier as they were not squeezed through filters, an event that could lead to stripping of surface proteins or even cell death and make them more vulnerable to complement attack. Thus, further studies are needed to systematically evaluate both methods of merozoite isolation in conjunction with merozoite infectivity, and complement activation level in the liquid phases and in the invasion assays.

## Complement in the immunity to asexual erythrocytic stages

Variant‐specific immunity to *P. falciparum* IE surface antigens is acquired in an age‐dependent manner [55, 56, 57] and associated with protection from clinical disease [55, 58]. Thus, it is expected that anti‐VSA antibodies could mediate both *P. falciparum* IE cell lysis and complement‐dependent phagocytosis of IE. Early work showed that *P. falciparum* IE is resistant to complement‐mediated lysis even though complement components were fixed on the IE surface [59, 60]. However, complement fixation on IE can still lead to complement‐dependent phagocytosis. In fact, it was demonstrated that phagocytosis of *P. falciparum* IE by macrophages and neutrophils in the presence of immune serum was enhanced when functional complement was present [87, 88]. However, no recent data are available on the role of complement in immunity against *P. falciparum* IE even though anti‐VSA antibodies can indeed react with the IE surface, agglutinate *P. falciparum* IE *in* *vitro* [55], and associate with protection from clinical disease [55, 58]. Thus, further investigations are needed to better understand the relationship between anti‐VSA antibodies, complement, and protection from malaria.

## Complement in the immunity to sexual stages

Gametocytes, female and male forms, of the *Plasmodium* parasites are formed in the human host through developmental switch from asexual to sexual forms of the parasite. Malaria transmission depends on successful completion of the sexual *Plasmodium* cycle in the mosquitoes that is initiated by gametocytes taken with a blood meal from the human host. Interrupting the sexual cycle at the fertilization stage, which involves gametocytes, in the mosquito midgut is recognized as a potential strategy for the control of malaria transmission. Indeed, a major surface antigen on the gametocyte surface, Pfs230, was shown to be target for transmission‐blocking monoclonal [89, 90, 91, 92] and polyclonal [93, 94] antibodies in standard membrane feeding assays. The antibody‐mediated transmission‐blocking activity of monoclonal and polyclonal antibodies was also shown to be complement‐dependent [89, 90, 91, 92, 93]. The monoclonal antibodies were of the mouse complement‐fixing subclass IgG2a [90, 92, 95]. Moreover, naturally acquired antibodies have been shown to recognize Pfs230 [96, 97]. Additionally, these antibodies had complement‐mediated transmission‐blocking activity against *P. falciparum* gametes and the activity was associated with the presence of antibodies specific to Pfs230 that were predominantly of the complement‐fixing type human IgG1 and IgG3 subclasses [96]. On the contrary, recent work has demonstrated that depleting naturally acquired antibodies from Pfs230‐specific antibodies retained high‐level complement‐independent transmission‐blocking activity, suggesting that antibodies to other gametocyte antigens were also involved [98].

## Complement in vaccine‐induced protection against malaria

Activation of the complement cascade by immunoglobulins plays a major role in the host defense against pathogens. Ranking complement activation by different IgG subclasses has indicated that IgG1 and IgG3 are the most potent subclasses in mediating complement activation. IgM is also a very potent complement activator. Thus, complement‐mediated protection against malaria induced by vaccines requires specific class or subclass of immunoglobulins to be induced by a given vaccine. The most advanced malaria vaccine RTS,S, based on CSP, was shown to induce high levels of IgG antibodies that were associated with protection against clinical malaria in RTS,S vaccine trials [16, 99]. It was further reported that vaccination of infants and young children with RTS,S induced IgG1 and IgG3 responses that correlated with protection, implying that complement might be involved [99]. IgG1 and IgG3 are also inducers of other Fc‐mediated effector functions, such as antibody‐dependent cellular cytotoxicity and phagocytosis. More recently, vaccination with RTS,S was shown to elicit anti‐CSP antibodies that were mostly IgG1. These antibodies fixed complement and were short‐lived and mirrored the decline in the RTS,S efficacy presented by the study cohort [21].

Whole sporozoite‐based vaccination also induced sporozoite‐specific IgG and IgM with complement‐fixing capacities. They induced sporozoite lysis and inhibited hepatocyte invasion [26]. Similarly, whole sporozoite‐based (Sanaria PfSPZ vaccine) vaccination was also shown to elicit long‐lived IgM class invasion‐inhibitory and complement‐fixing antibodies [27].

Earlier work by McCoy *et al*. [10] showed that in mice immunization with PfCSP elicited antibodies that targeted *P. falciparum* sporozoites and activated the classical pathway of complement resulting in cell death, although the immunoglobulin subclass identified in the study was mouse IgG1 known to be a poor complement activator [10]. Together, these results suggest that complement‐fixing antibodies play an important role in vaccine‐induced immunity.

Despite the partial success of the RTS,S and whole sporozoite vaccines, it has to be noted that the malaria vaccine development has been fairly short‐sighted with regard to the functions of the vaccine antigens and the respective antibodies induced. Immunoglobulin classes and IgG subclasses and invasion tests are not sufficient to monitor or screen most suitable and functionally efficient vaccine antigens. It is simply not enough just to monitor levels of antibodies produced. It is more important to know what they are doing. Which functions they are blocking if any. Different stages of the malaria parasites have multiple stage‐specific functions that could be addressed by the vaccines. Complement resistance is one of them. Another aspect not fully considered is the reasons behind species specificity of malaria parasites. Understanding this would already provide a better background for vaccine development.

## Conclusions and Perspectives

Recent data showed that *P. falciparum* parasite binds host complement regulatory proteins such as FH and C1‐INH to its surface to mimic the mechanisms that the host uses to escape complement‐mediated killing [19, 20]. Nonetheless, the surfaces of the *P. falciparum* developmental stages that evade complement activation were shown to be targets for complement‐fixing antibodies that were elicited naturally or *via* vaccination [25, 26, 27, 82]. The complement‐fixing activity of these antibodies was also reported to associate with protection in some studies [25, 82]. These findings suggest that the complement evasion mechanisms can be neutralized and overcome. One way to achieve this would be through repeated infections, in which sufficient neutralizing antibodies are mounted against the evasion molecules. Overcoming complement evasion mechanisms could also be achieved *via* vaccination. It is possible that the immune response against the vaccine candidate, usually a surface protein, may induce a quantity of complement‐fixing antibodies adequate for overloading the capacity of the bound regulator, thereby disabling the evasion mechanism. If the vaccine candidate is also an evasion molecule, an antibody against it would both neutralize its binding site and, if the neutralizing antibodies are of the complement‐fixing types (IgG1 and IgG3), also activate the complement on the parasite surface *via* the classical pathway. Thus, parasite antigens that can elicit complement evasion‐neutralizing antibodies with complement‐fixing abilities could already be a step forward in future malaria vaccine development.

Successful examples of this strategy are the meningococcal FHbp, which are now components of Bexsero® (Novartis) and Trumenba® (Pfizer) vaccines, recently approved against group B meningococcus [32]. FHbp was discovered in a systematic meningococcal genome‐wide search for immunogenic and protective bacterial antigens [11]. It was only later found that the exceptional activity of FHbp was based on its FH‐binding activity [40]. In addition to activating complement, FHbp‐specific antibodies block binding of FH and hence increase bacterial susceptibility to killing *via* the alternative pathway [42].

As we have noted earlier [12], the use of FH‐binding proteins as vaccines should consider the possibility that they could bind to their ligands, like FH, in individuals being vaccinated. This interaction may obscure potentially protective epitopes on the vaccine antigen and reduce antibody responses because of reduced complement activation usually needed for antigen delivery to lymph nodes. Although these possible interactions did not appreciably appear to hamper the immunogenicity of the FHbp in meningococcal vaccines, recent studies have shown that FHbp with impaired FH binding, by single amino acid substitution, elicited enhanced bactericidal antibody responses in human FH‐transgenic mice [13]. In a recent study, eliminating the FH‐binding ability of another bacterial complement evasion molecule, *B. burgdorferi* CspZ, also resulted in greater bactericidal antibody titers in mice vaccinated with modified CspZ than the nonmodified version [14] Thus, discovering the appropriate *Plasmodium* complement evasion molecules and mutating them to eliminate the binding ability to their targets may be needed to develop functionally active vaccines with sufficient immunogenicity and efficacy.

## Funding

Research funding to SM and AK was provided by the Helsinki University Hospital Funds (TYH2019311), the Sigrid Jusélius Foundation (4705080), the Academy of Finland (1323237), and the Jane and Aatos Erkko Foundation (4706167). PKK was supported through the DELTAS Africa Initiative [DEL‐15‐003]. The DELTAS Africa Initiative is an independent funding scheme of the African Academy of Sciences (AAS)'s Alliance for Accelerating Excellence in Science in Africa (AESA) and supported by the New Partnership for Africa's Development Planning and Coordinating Agency (NEPAD Agency) with funding from the Wellcome Trust [107769/Z/10/Z] and the UK Government. The views expressed in this publication are those of the authors and not necessarily those of AAS, NEPAD Agency, Wellcome Trust, or the UK Government.
